# CXCR4 Is a Potential Target for Anti-HIV Gene Therapy

**DOI:** 10.3390/ijms25021187

**Published:** 2024-01-18

**Authors:** Appolinaria K. Prokopovich, Irina S. Litvinova, Alexandra E. Zubkova, Dmitry V. Yudkin

**Affiliations:** 1 State Research Center of Virology and Biotechnology “Vector”, Federal Service for Surveillance on Consumer Rights Protection and Human Well-Being (FBRI SRC VB “Vector”, Rospotrebnadzor), 630559 Koltsovo, Russia; prokopovich_ak@vector.nsc.ru (A.K.P.); litvinova_is@vector.nsc.ru (I.S.L.); zubkova_ae@vector.nsc.ru (A.E.Z.); 2 Department of Natural Sciences, Novosibirsk State University, Pirogova 2, 630090 Novosibirsk, Russia

**Keywords:** CXCR4, HIV, gp120, gene therapy

## Abstract

The human immunodeficiency virus (HIV) epidemic is a global issue. The estimated number of people with HIV is 39,000,000 to date. Antiviral therapy is the primary approach to treat the infection. However, it does not allow for a complete elimination of the pathogen. The advances in modern gene therapy methods open up new possibilities of effective therapy. One of these areas of possibility is the development of technologies to prevent virus penetration into the cell. Currently, a number of technologies aimed at either the prevention of virus binding to the CCR5 coreceptor or its knockout are undergoing various stages of clinical trials. Since HIV can also utilize the CXCR4 coreceptor, technologies to modify this receptor are also required. Standard knockout of *CXCR4* is impossible due to its physiological significance. This review presents an analysis of interactions between individual amino acids in CXCR4 and physiological ligands and HIV gp120. It also discusses potential targets for gene therapy approaches aimed at modifying the coreceptor.

## 1. Introduction

HIV belongs to the Retroviridae family and causes acquired immunodeficiency syndrome (AIDS). According to the World Health Organization (WHO), the estimated number of people infected with HIV-1 in 2022 is 39,000,000; more than one million new infection cases are reported every year [1]. There are two main HIV species: HIV-1 and HIV-2. The immune response to HIV-2 is more effective, while HIV-2 is characterized by lower transmissibility, a lower risk of progression to AIDS, and higher conservation of the virus envelope glycoprotein [2,3].

HIV-1 replicates predominantly in CD4+ T cells, resulting in T cell depletion, which is the main cause of immune failure. Virus fusion with target cells is facilitated by binding to the CD4 receptor and one or two coreceptors. Depending on cell tropism, the virus utilizes CXCR4 (X4-tropic), CCR5 (R5-tropic), or two transmembrane proteins (X4/R5-tropic) as coreceptors [4]. The most common transmitted form is R5-tropic, and as infection progresses, the R5-tropic virus may change tropism to X4-tropic through acquisition of dual tropism to both CCR5 and CXCR4 [5,6].

Despite significant advances in antiretroviral therapy (ART), the HIV pandemic persists, and the infection remains incurable [1,7]. According to the WHO, over half a million people died from HIV-related causes in a year. Gene therapy, which is being actively developed, can serve as an alternative to ART.

The developing gene therapy approaches are aimed at different stages of HIV infection: from preventing viral penetration into the cell to eradicating provirus from infected cells. Additionally, therapies can be based on chimeric antigen receptor (CAR)-T technologies or neutralizing nanobodies and are aimed at virus destruction or infected cells’ destruction [8,9].

Strategies aimed at viral life cycle blocking are based on RNA interference (RNAi), programmed nucleases, aptamers, intrabodies or ribozymes. For example, these approaches can inhibit HIV-1 replication by silencing some HIV regulatory genes or preventing viral penetration into the cell by inhibiting their interaction with coreceptors [10]. Depending on the strategy, ex vivo or in vivo therapy can be used. In case of an ex vivo strategy, patients’ cells (for example, hematopoietic stem cells) should be modified in vitro and autologously transplanted. In case of in vivo therapy, the drug is administered into the targeted organ or tissue [11,12,13].

In this paper, we will describe in detail the approaches aimed at the prevention of HIV-1 binding to the cell. For example, natural mutation Δ32 in *CCR5* gene leads to the absence of CCR5 coreceptor on the cell surface and carriers of this mutation are sustainable to HIV-1 infection [14,15,16]. Similarly, therapeutic approaches preventing binding to CCR5 are aimed at *CCR5* knockout. Today, there are several approaches in clinical trials. These are based on short hairpin RNA (shRNA) (NCT02797470, NCT02343666, NCT02337985, NCT01961063, NCT01153646, NCT00569985), zinc-finger nucleases (ZFNs) for CCR5 knockout (NCT02500849, NCT02225665, NCT02388594, NCT01044654, NCT01252641, NCT00842634, NCT03666871, NCT03617198), and CRISPR-Cas9 for CCR5 knockout (NCT03164135, NCT05144386, NCT05143307) [17,18,19].

The developing therapeutic approaches aimed at the prevention of virus binding to CXCR4 are based on CXCR4 inhibition by peptides like AMD3100, natural ligand-like molecules, small interfering RNAs (siRNAs) or nanobodies [20,21,22,23,24]. Another way is the usage of chimeric coreceptor binded with viral heptad repeat-2 [25]. There is not a CXCR4-based strategy in clinical trial today. The main problem is the very important physiological role of this protein [26,27,28,29].

The CXCR4 coreceptor is expressed in a wide range of both embryonic and somatic tissues and organs [30,31,32]. Two receptor isoforms produced as a result of alternative pre-mRNA splicing are known: the highly expressed B variant, and the long unspliced A variant [33]. The interaction of CXCR4 with extracellular ligands such as CXCL12, extracellular ubiquitin (eUb), and macrophage migration inhibitory factor (MIF) triggers intracellular signaling, thus facilitating the migration of hematopoietic stem cells into the bloodstream [26,34], as well as immune cell maturation and activation [27,32,35,36]. The effect of these interactions on the cell cycle has also been shown [37,38]. *Cxcl12* and *Cxcr4* knockout mice were shown to have poor perinatal survival, while the surviving mice are characterized by impaired development of the brain, lung, heart, and the majority of other organs. In addition, these mice exhibited immune deficiency due to disrupted lymphocyte development [26,27,28,29]. Thus, complete *CXCR4* knockout cannot be used to prevent HIV infection, as in the case of *CCR5*, because it would have an extremely severe effect on the organism. Similarly, due to the same reasons, a specific CXCR4 inhibitor—AMD3100—cannot be used for therapy [39]. In contrast, maraviroc—a CCR5-specific inhibitor—is approved for use in Europe and the USA [40].

The substitutions that have been studied to date do not result in a significant decrease in the sensitivity of *CXCR4* mutant cells to HIV compared to that of wild-type cells [41]. In this regard, it is necessary to study other amino acids as potential targets for gene therapy. An analysis of the interaction between natural ligands and the V3 loop can help in identifying potential gene therapy mutations that inhibit binding to HIV without affecting the physiological function of CXCR4.

Finally, none of the developed strategies can be effective alone, and the further development of anti-HIV therapeutic approaches is needed for effective virus elimination using a combination of strategies with different targets.

## 2. Interaction of CXCR4 with Ligands

**Figure 1:**
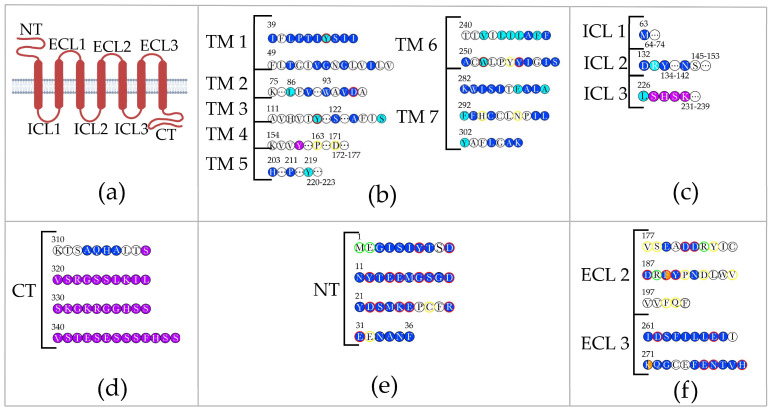
Schematic representation of the CXCR4 receptor and the involvement of its amino acids’ residues in the interaction with ligands and HIV gp120. (**a**)—Schematic representation of CXCR4 and its domains: NT—N-terminal domain, TM—transmembrane domain, ICL—intracellular domain, ECL—extracellular domain, CT—C-terminal domain; (**b**)—TM primary structure; (**c**)—primary structures of ICL1, 2, and 3; (**d**)—CT primary structure; (**e**)—N-terminal domain; (**f**) primary structures of ECL2 and 3. Highlighted amino acids are involved in ligand interaction: dark blue—interaction with CXCL12; orange—interaction with eUb; light blue—interaction with the G protein and signal transmission through the G-dependent pathway; purple—signal transmission through the G-independent pathway. Amino acids required for binding to HIV gp120 are indicated in colored contours: red—amino acids required for both receptor and coreceptor activity; yellow—amino acids required only for binding to the V3 loop of the HIV gp120, which are considered potent targets for therapeutic mutation; green—amino acids required for binding to the V3 loop of the HIV gp120, which have been identified by us as the most potential gene therapy targets.

The chemokine receptor CXCR4 belongs to the family of rhodopsin-like G protein-coupled receptors (GPCRs); it has a common transmembrane structure shared by all GPCRs. The extracellular profile of CXCR4 is determined by the negatively charged N-terminus, which is involved in ligand binding, and three extracellular loops (ECLs), which connect the transmembrane domains (TMs). The internal profile of the receptor includes intracellular loops (ICLs) and the C-terminus, which are responsible for cell signaling [42] (Figure 1a).

CXCR4 activation triggers numerous signaling pathways associated with gene transcription regulation, cell migration, and adhesion [42]. These pathways can be conditionally classified as either G protein-dependent or -independent pathways.

The G-dependent pathway involves signaling from the extracellular environment into the cell through the binding of the G protein to the receptor C-terminus. An analysis of conserved and non-conserved mutations revealed amino acids involved in signal transduction through CXCR4 in a G-dependent manner. Non-conserved substitutions of amino acids Tyr45, Trp86, Tyr116, and Glu288 (Figure 1b,c) are known to disrupt signal initiation. Substitutions at positions Val242, Leu244, Ile245, Leu246, Phe248, Trp252, Ala291, and Phe292 (Figure 1b) affect signal propagation through the receptor, while amino acids Ser131, Arg134, Tyr219, Leu226, and Tyr302 are responsible for G protein binding and activation (Figure 1b) [43].

The G-independent pathway is a signaling pathway through GPCRs that does not involve G proteins. This pathway ultimately leads to the transcriptional activation of a series of cytokine-responsive genes. In the case of CXCR4, the G-independent pathway is ensured through JAK/STAT pathway. After CXCR4 binds to the extracellular ligand, in addition to the G protein activation, JAKs phosphorylates Tyr residues in the internal domains of receptor. The phosphorylated receptor, when complexed with these kinases, recruits and activates STAT transcription factors [33]. Specific amino acids in CXCR4 required for JAK-mediated receptor phosphorylation have not been identified yet. However, non-conserved substitutions of amino acids in the SHSK motif on the third ICL and Tyr157 (Figure 1b,c) are known to prevent the activation of STAT factors [44].

In addition to JAK/STAT signaling, G-independent regulation through CXCR4 can be associated with beta-arrestin. Beta-arrestin is known to activate transcription and cell migration through intracellular mediators [45]. Furthermore, the interaction of CXCR4 with beta-arrestin is mediated through the phosphorylation of Ser and Thr in the third ICL and the receptor C-terminus. This phosphorylation is one of the outcomes of the G-dependent pathway. Beta-arrestin is complexed, and the receptor not only activates additional signaling pathways but also desensitizes CXCR4 to the ongoing effects of ligands, thus terminating further signal transmission through the G-dependent pathway. As a result, beta-arrestin provides a negative feedback mechanism in G-dependent signaling. In addition, the association of CXCR4 with beta-arrestin ensures further receptor internalization [45].

Impaired beta-arrestin-mediated G protein activation is determined by C-terminal amino acids in CXCR4. The deletion of a 34-amino-acid sequence in the C-terminus, which contains potential phosphorylation sites (Figure 1d), enhances the receptor G-dependent activity [46]. Moreover, the deletion of the SHSK motif in the third ICL (Figure 1c) causes pronounced internalization in the presence of beta-arrestin. In other words, the presence of the SHSK motif apparently stabilizes CXCR4 on the cell membrane by regulating its desensitization [47].

Thus, the G protein-dependent and -independent signaling pathways are closely connected and provide parallel regulation upon CXCR4 activation with the same extracellular ligands, such as the chemokine CXCL12.

The receptor N-terminus is required for the CXCL12–CXCR4 complex formation. A and B isoforms of CXCR4, which differ in the N-terminus sequence, are activated in response to chemokines with the same effectiveness rate [48]. In silico substitution of the first 27 amino acids in CXCR4 with the corresponding region in its homolog CXCR2 impaired binding with CXCL12, while the substitution of all amino acids in the N-terminus rendered this interaction completely impossible [49]. Conserved and non-conserved substitutions of individual amino acids demonstrated that Asp97, Asp262, and His281 in the CXCR4 N-terminus (Figure 1e), as well as amino acids Asp187, Phe189, Asn192, and Leu267 in the ECLs (Figure 1f), are required for binding to the CXCL12 N-terminus [43]. Moreover, the results of numerous in vitro and in silico studies of chimeric and mutant CXCR4 forms have shown that amino acids in all TMs, except for domain 4, as well as in ECLs 2 and 3, are required for binding to CXCL12 (Figure 1b,f) and G-dependent signaling in the cell [43,50,51,52,53].

Studies on CXCR4 complex formation with extracellular ligands show that the activation of CXCL12 and eUb triggers similar G-dependent responses. However, the mechanism of CXCR4 interaction with eUb differs from that of chemokine activation. The receptor N-terminus is not involved in the CXCR4–eUb complex formation: nuclear magnetic resonance (NMR) spectroscopy of the CXCR4 N-terminus (1–38) and analysis of signal transmission in the presence of N-terminal-specific antibodies revealed the absence of binding sites for CXCR4 and eUb in this region [54]. However, in silico studies proposed other amino acids in CXCR4 that may be involved in the CXCR4–eUb complex formation: Phe29, Phe189, and Lys271 (Figure 1e,f) [55].

There is evidence of the role of MIF as an extracellular ligand for CXCR4. MIF was shown to interact with a peptide that presents a truncated domain of the ECL2 (182–199) (Figure 1f). However, no specific binding sites have been identified yet. At the same time, the data indicating a decrease in chemokine-dependent activity of CXCR4 due to an increase in the MIF level suggest that there is competition between MIF and CXCL12 for binding and signaling through CXCR4. This, in turn, suggests the presence of common binding sites [37].

CXCR4 is crucial for normal cell functioning due to its ability to mediate G protein-dependent and -independent signaling by binding to specific extracellular ligands. Physiologically important sites in CXCR4 for binding with other factors are located on the receptor N- and C-termini, in ECLs 2 and 3, ICLs 2 and 3, and in almost all TMs (Figure 1c–f). Such a wide range of interactions complicates the search for modification targets that can be used to inhibit binding to HIV. However, an analysis of sites involved in effective HIV infection through CXCR4 will enable the identification of potential targets for gene-editing therapy.

## 3. Interaction of CXCR4 with the V3 Loop in HIV gp120

The interaction of HIV with coreceptors is determined by binding sites on the viral V3 loop. The V3 loop sequence is variable in wild-type virus populations, as it differs between strains with different tropism for coreceptors CXCR4 and CCR5 [56,57]. In silico studies showed that amino acids 8–26 in the HIV V3 loop are integrated inward of CXCR4, while the first seven amino acids (27–35) in the V3 loop are located next to the CXCR4 N-terminus [58,59]. In addition, studies using chimeras obtained based on CXCR4 and CXCR2 when infecting cells with a recombinant virus demonstrated that the N-terminus, ECL2, and ECL3 of CXCR4 play an important role in coreceptor activity (Figure 1e,f) [52]. Truncation of the CXCR4 cytoplasmic tail and mutation of the conserved DRY motif (Figure 1c) in the second ICL had no effect on the coreceptor function [52,60].

An in vitro single substitution at Glu2, Asp10, Glu14, Glu15, Asp20, Asp22, Ser23, Lys25, Glu26, Cys28, and Glu32 in the N-terminus domain, as well as simultaneous substitution of Tyr7 and Tyr21 to Ala in CXCR4, resulted in decreased coreceptor activity (Figure 1e). The effect of single substitutions was demonstrated in experiments on infecting cells expressing a mutant CXCR4 form with a lentivirus pseudotyped with the HIV-1 env; the effect of simultaneous substitution was studied in experiments on cell infection with HIV-1 [51,61]. These results correlate with in silico studies. A computer modeling study described the interaction of Met1, Gly17, Ser18, Phe29, and Arg30 in CXCR4 with the V3 loop in HIV gp120 through hydrogen bonding (Figure 1e) [59].

Various mutations leading to substitutions of amino acids in the second ECL at positions Arg183, Arg188, and Asp193 with other amino acids, as well as Tyr184 to Ala substitution, either decreased or inhibited coreceptor binding to HIV (Figure 1f) [62]. The substitution of Asp182, Asp187, Phe189, and Pro191 with Ala resulted in a decreased binding effectiveness of CXCR4 to the HIV-1 Env expressed on the cell surface (Figure 1f) [63]. In silico studies demonstrated that Val177, Ser178, Tyr190, Phe199, Asp262, and Glu268 in ECLs 2 and 3 are also important for the interaction with the V3 loop (Figure 1f). In particular, Glu268 participates in the direct formation of the CXCR4–V3 loop complex, while Asp262 stabilizes it [59].

In addition to amino acids located in ECLs, amino acids in TMs are also required for proper binding of the receptor to HIV. They typically determine the stability of the complex with the V3 loop. An analysis of amino acids in TMs 1, 2, and 4 using recombinant viruses showed that Phe87 forms a π–π bond with CXCR4 Tyr116, which directly interacts with HIV V3 (Figure 1b). Trp161 and Pro163 stabilize the spiral configuration of TM 4 (Figure 1f). Asp171 in CXCR4 is considered one of the crucial amino acids involved in HIV coreceptor activity, since its substitution with Ala reduces the possibility of coreceptor binding to HIV by more than 60%. The substitution of Tyr45, Asp97, and His79 decreases the coreceptor activity, although to a lesser extent (Figure 1b) [52,60]. Amino acids Gln200, Tyr255, Tyr256, and Glu288 in TMs 5, 6, and 7 participate in a complex formation with the V3 loop, while Trp252 stabilizes it. The contribution of His294 and Asn298 is likely associated with the activation and maintenance of the CXCR4 intermolecular interactions that mediate binding to HIV (Figure 1b) [59].

The presence of CXCR4-tropic strains in the body indicates disease progression. X-tropic strains are associated with a more rapid failure of the immune system since they infect both naive and memory T cells [64]. On the other hand, X4-tropic virus appearance is based on CCR5-positive cells’ depletion. It leads to positive selection of X4-tropic viruses. Additionally, a change in tropism can be based on R5-tropic specific humoral immune response [65]. Moreover, a suppressed humoral immune response allows the X4-tropic virus to dominate on the R5-tropic virus [66,67]. Amino acids that are important for CXCR4 binding to the V3 loop are located in almost all ECLs and TMs except for ECL 1 and TM 5 (Figure 1b,f). The majority of these amino acids are important for proper coreceptor functioning. However, some residues are solely involved in the binding of CXCR4 to HIV-1 V3. These amino acids can be considered potential targets for gene therapy.

## 4. Discussion

For cell entrance, HIV uses the glycoprotein complex Env, part of which is the gp120 glycoprotein. As a result of its interaction with CD4, the glycoprotein undergoes conformational changes, which leads to its binding to one of the following coreceptors: CCR5 or CXCR4. The binding of gp120 to coreceptors leads to the proximity of the cell membrane and the virion membrane with their subsequent fusion [68,69,70].

In this study, we conducted an analysis of the available literature on the interaction between CXCR4 and natural ligands, as well as of the HIV-1 V3 loop. Not all amino acids coincide, but the majority of them involved in complexing with HIV gp120 and involved in complexing with natural ligands do. However, some studies show that the ability of CXCR4 to function as an HIV coreceptor does not depend on its ability to bind chemokines [52]. Hence, the mismatched amino acids can be considered targets for gene therapy.

Amino acids at positions 3–26 and 29–31 in the N-terminus of CXCR4 are required for binding both natural ligands and HIV V3 (Figure 1e). The use of these residues as targets for gene therapy can impair receptor function. Met1, Glu2, and Cys28 specifically bind to HIV V3 (Figure 1e). It was also shown that the B isoform of CXCR4, which has a shorter but more negatively charged N-terminus compared to the A isoform, enables a more efficient virion penetration into the target cells. At the same time, both isoforms exhibit the same chemotactic activity towards CXCL12 [48,71]. Therefore, amino acids located at the beginning of the N-terminal domain are of particular interest as targets for gene therapy (Figure 1e). Val177, Ser178, Arg183, Tyr184, Arg188, Pro191, Asp193, Val196, Phe199, and Gln200 in the second ECL were identified as residues important for exclusive coreceptor binding to HIV (Figure 1f). Hence, these amino acids can also serve as targets for gene therapy.

Residues located in TMs are important for stabilizing the complex with the V3 loop and signaling into the cell through the G-dependent pathway. Phe87, Tyr116, Pro163, Asp171, Trp252, Tyr255, His294, and Asn298 are not associated with the physiological role of CXCR4 and are only involved in coreceptor function (Figure 1b). This implies that they have the potential to be used as targets for gene therapy.

Furthermore, future studies should take into account the high variability of the HIV V3 loop. Data show that the following amino acids are crucial for the recognition of CXCR4 regions by the HIV V3 loop: Arg9, Lys10, Arg11, Leu14, Arg18, Trp20, Tyr21, Arg31, and Lys32. In addition, one or few of the residues in the V3 loop at positions 11, 24, and 25 should have a positive charge [56]. These findings suggest that the V3 loop exhibits high, but limited, variability. The most conserved V3 loop regions bind to Met1, Glu2, Asp10, Glu14, Glu17, Ser18, Asp20, Phe29, Arg30, Arg183, Arg188, Tyr190, Asp193, Glu277, Asn278, and His281 in CXCR4 [59]. Among these, Met1, Glu2, Arg183, Arg188, and Asp193 specifically bind to the V3 loop (Figure 1e,f). Apparently, these residues will serve as the most effective targets for gene therapy in future studies.

We suggest that the most effective gene therapy strategy is to introduce targeted mutations in sequences encoding potential targets, which will result in amino acid substitutions. It is impossible to predict in advance which substitutions will be necessary to achieve a gene therapy effect. For this reason, a panel of mutations should be studied in order to identify the target ones.

Mutagenesis methods for all modern therapeutic approaches should be developed and studied. For example, today, ex vivo modification of hematopoietic stem cells and their autologous transplantation looks promising. This approach is now in use to treat some other disorders (NCT03041324, NCT03655678, NCT03745287) [72,73]. Ex vivo modification allows researchers to detect off-target effects, which is impossible in the case of in vivo modifications. But ex vivo therapy needs many more stages, including cell collection, cultivation, selection and transplantation [74,75]. Moreover, HIV not only infects blood cells but also glial, neuronal, astrocytic, epithelial and endothelial cells in different organs. Therefore, in vivo therapies’ development is required.

Different genome-editing methods are now in use in laboratory and clinical trials, and can be used in future CXCR4 modification. For example, single-amino-acid substitution can be reached with use of CRISPR-Prime Editing or through editing with a DNA donor for homology recombination. These approaches have off-target effects and should be used ex vivo. For example, an approach with homology recombination was used for chronic granulomatous disease treatment in mouse models, but it is not in clinical trials now because of the toxicity of the exogenous oligonucleotide templates, the heterogenous pool of edited cells, and its low effectivity in vivo [76,77,78].

The active development of genome-editing approaches and of their effectiveness leads to significant progress in the field of gene therapy for hereditary and infectious diseases. Future developments of protein editors, and the development of recombinant editors will allow for a decrease in the off-target effect to almost zero and increase the effectivity of editing. In the very near future, we will see the latest gene therapy strategies based on the latest genome editors, which will allow researchers to make single substitutions in DNA [79,80]. The study of targets for mutations in CXCR4, presented in this review, and the development of genome-editing technologies may bring us closer to creating optimal HIV treatment strategies.

## Data Availability

Not applicable.
